# Factors that influence the decision to vape among Indigenous youth

**DOI:** 10.1186/s12889-022-13095-y

**Published:** 2022-04-02

**Authors:** Laura L. Struik, Saige-Taylor Werstuik, Alyssa Sundstrom, Sarah Dow-Fleisner, Shelly Ben-David

**Affiliations:** 1 Faculty of Health and Social Development, School of Nursing, The University of British Columbia, Okanagan Campus, Syilx Okanagan Nation Territory, 1147 Research Road ART 140, Kelowna, BC V1V1V7 Canada; 2 Westbank First Nation, West Kelowna, British Columbia Canada

**Keywords:** E-cigarettes, Indigenous youth, Qualitative research

## Abstract

**Background:**

The use of e-cigarettes (vaping) among Indigenous youth is much higher than that of their non-Indigenous counterparts, which has raised the concerns of various Indigenous scholars and communities. To better understand the most salient constructs that influence Indigenous youth decision-making around vaping, we co-created a qualitative research study with a Syilx First Nation community that was guided by the Unified Theory of Behavior (UTB).

**Methods:**

Through semi-structured interviews and a sharing circle, we gathered the perspectives and experiences of 16 Syilx youth in British Columbia, Canada. After an initial collaborative coding and training session, the interviews were transcribed and coded by Indigenous peer researchers using Nvivo. Through both directed and conventional qualitative content analysis methods, the final conceptual framework was collaboratively developed.

**Results:**

Syilx youth reported that vaping decision-making is underpinned by colonialism, and the historical disproportionate impact of the tobacco industry. The youth spoke to several individual determinants that influence intentions to vape (e.g., vaping helps you cope) and to not vape (e.g., family and community connectedness), and determinants that translate intentions to vape to decision to vape (e.g., access to vaping), and to not vape (e.g., access to trusted adults and support from the band). The youth suggested that prevention efforts must be informed by an understanding of why Indigenous youth vape and what strengthens their resolve to not vape.

**Conclusions:**

Vaping decision-making among Indigenous youth is underpinned by their cultures, contexts, and histories. To effectively address vaping among Indigenous youth, continued engagement of Indigenous youth in planning, developing, implementing, and evaluating both prevention and policies efforts is a necessity.

## Introduction

### Background

Youth have been the primary target for tobacco alternatives, particularly e-cigarettes [1], which is underscored by their high rates of use. In 2020, 14% of Canadian youth ages 15–19 vaped in the past 30 days, and over a third (35%) reported ever trying it [2]. While young adults ages 20–24 share similar statistics with past 30-day use and ever trying at 13 and 43% respectively, adults over the age of 25 had significantly lower rates, at 3 and 13% respectively [2]. Additionally concerning is emerging evidence that Indigenous populations of youth in North America have higher usage rates and are at higher risk for e-cigarette use compared to their non-Indigenous counterparts [3, 4]. For example, in a study that examined e-cigarette behaviour among a national sample of over 14,000 youth in the United States, the authors found that compared to non-Indigenous youth, American Indian/Alaska Native students had the highest prevalence rates among dual users (using both combustible cigarettes and e-cigarettes) (16.2%) (RR: 2.10), and e-cigarette only users (11.3%) (RR: 1.70) [3]. Researchers in British Columbia, Canada, examined vaping across five communities and stated that vaping was found to be a significant issue in all of these communities [4]. Given that youth vaping is associated with increased risk for tobacco and other substance use, mental health problems, pulmonary and cardiovascular disease, and unintentional injuries [5], Indigenous youth are vulnerable to these adverse outcomes at a larger scale.

Indigenous populations continue to be a target by the tobacco industry for using tobacco alternatives, including e-cigarettes [4, 6, 7]. The disproportionate tobacco use and tobacco marketing towards Indigenous people can be viewed as colonization [4]. Colonization is the process of the assimilation of a people forced to adapt to the social, economic, and political protocols of a sovereign nation [8]. From an Indigenous perspective, colonization has been implemented through land displacement, resource extraction, assimilation, cultural appropriation and other aspects [9]. While some Indigenous groups use traditional tobacco for cultural ceremonies, this has been misappropriated by the tobacco industry through use of cultural imagery and concepts when marketing commercial tobacco [10], lending to significantly higher rates of tobacco use among Indigenous populations, including youth.

Indigenous people and scholars have been increasingly raising concerns around the tobacco industry’s purposeful interest in appropriating Indigenous people’s names and imagery to further tobacco sales and profits [4, 7, 11, 12]. As a result, leading Indigenous scholars have recently rejected support from the Phillip Morris-funded Foundation for a Smoke-Free World for Indigenous Health Research, stating that this support is contrary to the health and well-being agendas of Indigenous people [6]. Researchers and Indigenous scholars are calling for more comprehensive and responsive efforts to narrow the disproportionate tobacco marketing and use among Indigenous youth, which is now inclusive of e-cigarettes [3, 4, 7, 13].

In Canada, youth vaping prevention efforts not only remain sparse, but are also primarily designed to reach the general population of Canadian youth, like the national campaign, Consider the Consequences [14]. Researchers have recently implored the tobacco control community to stop rolling out generic tobacco control efforts in light of substantial evidence that tobacco use is underpinned by differential inequities experienced by different populations, including Indigenous populations [15]. In fact, studies have revealed robust correlations between affiliation and engagement with their culture and Indigenous youth well-being [16–19]. In this regard, it is recommended that Indigenous-based research is led by Indigenous people and includes Indigenous voices and methods of data collection and interpretation to build strength and resiliency through health promotion efforts, including tobacco control efforts, in the community [18]. In order to develop meaningful and tailored e-cigarette prevention initiatives for Indigenous youth, it is essential to first explore the unique factors that underpin their decision-making as it pertains to vaping, and their preferences for how these factors are addressed, which is the aim of this study.

### Conceptual framework

**Fig. 1:**
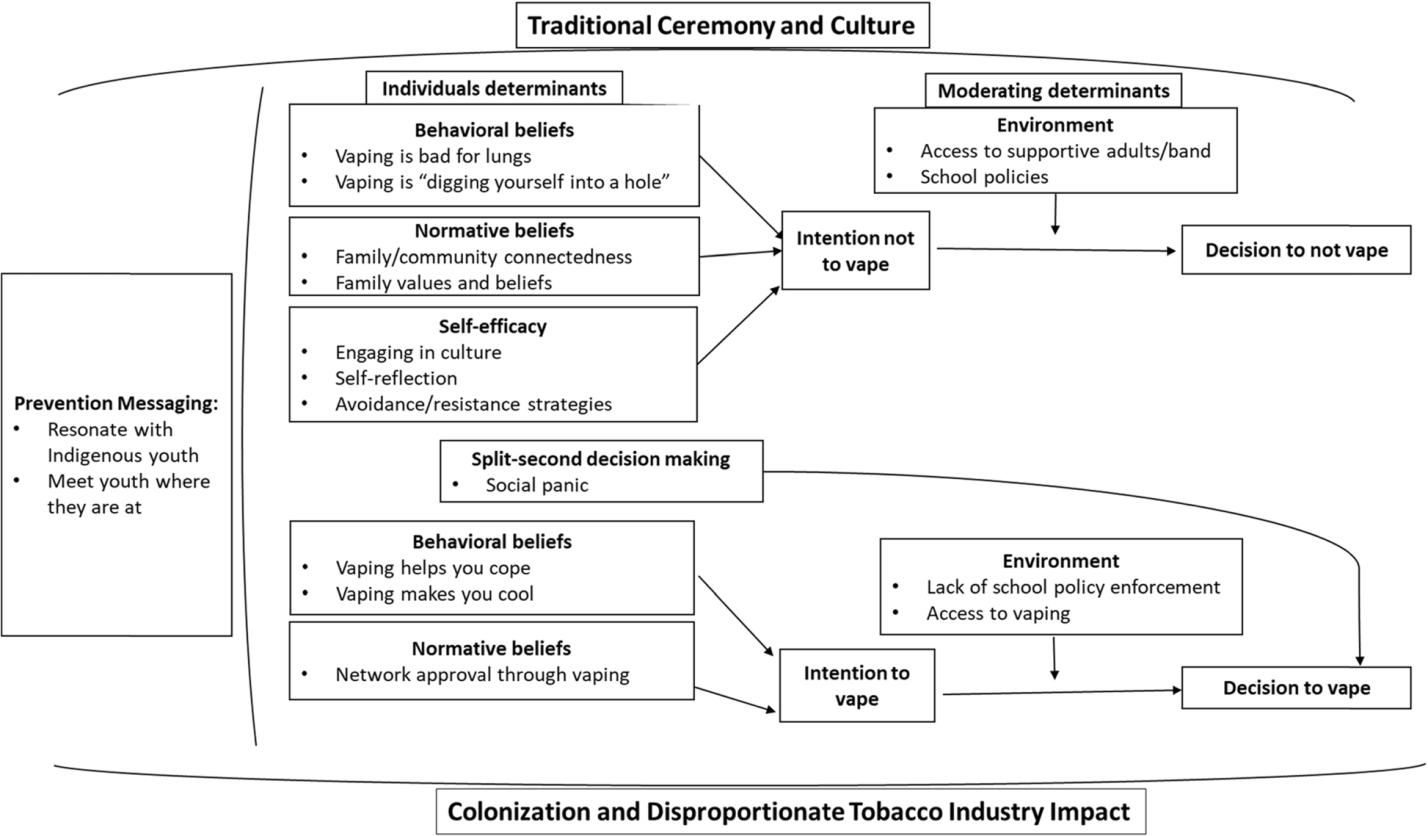
Conceptual Framework of Findings

Given the relationship between colonization and tobacco use among Indigenous youth, and the importance of culture to promote resiliency and positive health behaviors, this work is grounded in a culturally responsive approach. A culturally responsive approach prioritizes co-creation of knowledge development, and asking reflexive questions like, “How do the participants’ social and cultural identities inform their unique communication practices that must be acknowledged within this research?” [20]. In this study, the research plans, implementation, and interpretations were co-created with two trained Indigenous peer researchers (SW and AS) (both leaders at their First Nation community youth center). In this regard, Indigenous youth culture underpinned the research protocols and plans. Given that the focus of this research was to understand decision-making around vaping among Indigenous youth, we employed Unified Theory of Behavior to develop a theoretical and evidence-based understanding of vaping among Indigenous youth. This holistic theory [21–23] consists of five determinants of behaviour intention (behavioural beliefs, normative beliefs, self-concept, emotions, self-efficacy), and four determinants that impede/facilitate the translation of intention to actual behaviour (knowledge, environment, salience, and habitual processing), and one recently added construct (split-second decision making [23] (see Fig. 1). This framework has been recently utilized to understand help-seeking among youth and young adults with mental health problems [24–27]. Given the comprehensive nature of the UTB, use of this theory in the context of Indigenous culture and historical colonialism will enable a deep understanding of the most salient determinants that underpin Indigenous youth decision-making around vaping, ultimately informing the subsequent creation of more meaningful interventions that leverage factors promoting abstinence from vaping and address factors that lend to vaping behaviour. The purpose of this study therefore, was to explore determinants that influence the decision to vape among Indigenous youth.

## Methods

### Overview

This qualitative study employed Indigenous research methods (e.g., traditional methods of data collection, like sharing circles) [28] to understand decision-making around vaping among Indigenous youth. Indigenous research methods are described as methods that fully engage participants and incorporate experiential knowledge which align with qualitative research [28]. The methods of data collection must also be respectful of and include Indigenous protocols, values, and beliefs that are important to the specific community [29]. In this regard, the research protocols herein were co-developed and implemented by two trained Syilx peer researchers (SW and AS). In addition, all of the qualitative questions and questionnaire items asked of the participants were co-developed with the Indigenous peer researchers. This study also received a letter of support and approval by the Westbank First Nation Chief in addition to receiving Behavior Research Ethics Review Board approval (#H20–02312).

### Data collection

Two types of data collection were employed in this study: semi-structured interviews, and an Indigenous sharing circle [30]. All participants filled out a demographic questionnaire that asked about their demographic information (e.g., age, gender), social context (e.g., number of older peers), tobacco product use and exposure, and social media use. For the interviews, seven youth were recruited via social media ads posted by a third party consulting group (PH1 Research), as well as on the Westbank First Nation social media pages. All youth were directed to contact a Syilx peer researcher. Youth were eligible if they identified as Indigenous, were between the ages of 10 and 30 (in keeping with the Syilx definition of “youth”), and able to communicate in English. All eligible youth gave informed consent, and youth assent and parental consent in the case of those under the age of 16. Youth then filled out a brief demographic questionnaire, and participated in a ~ 45 min interview with a peer researcher where they answered questions about factors in relation to the UTB that influence their decision-making around vaping (see Table 1), as well as how their culture underpins their decision-making (e.g., “How do you see vaping in relation to Indigenous culture?”). Each participant received a $25 gift card to thank them for their time.

**Table 1:** UTB interview questions

UTB construct	Example questions
Behavioral beliefs	Why do youth vape?
Normative beliefs	Who in your life approves/disapproves of vaping?
Self-concept	How would you describe your peers who vape/don’t vape?
Emotions	What feelings emerge when you think about vaping?
Self-efficacy	What strategies do you use to manage social pressures/stressful life events?
Split-second	In what situations are you tempted/most likely to vape?
Knowledge	What knowledge do you have about vaping?
Environment	What makes it easy/difficult to vape?
Behavioral cues	What might be some triggers to vaping?
Habits	What habits might be associated with vaping?

The Syilx peer researchers led the conceptualization, planning, and organization of the sharing circle. Nine youth who had not previously participated in an interview were recruited through an Indigenous-led summer camp program to participate in the sharing circle. All eligible youth gave informed consent, and parental consent in the case of those under the age of 16. Youth then filled out a brief demographic questionnaire, and participated in a 60 min sharing circle session that opened with traditional smudging by an Indigenous peer researcher from the community, and then engaged in traditional methods of sharing. During the sharing circle, each participant passed around a ceremonial item (held by the person who is speaking) and expanded on the interview data, including their views on vaping among Indigenous youth, views on school policy, and ideas for prevention messaging. They then engaged in a brainstorming activity around prevention strategies. The session concluded with serving traditional food from their local community. Each participant received a $30 gift card and a small gift to thank them for their time.

### Data analysis

The interviews and the sharing circle were digitally recorded, transcribed by the research team, and then integrated into NVivo 1.3 (QSR International) qualitative data management software program for direct and conventional qualitative content analysis [31]. The first stage of analysis included collaborative coding of two interview transcripts among the first three authors (LS, SW, and AS) to develop the initial high-level codebook. The two peer researchers (SW and AS) then independently coded the remaining transcripts, noting nuances within the codes and consulting with the lead author as needed. Once complete, the lead author then reviewed the final coded set and worked with (SBD), an experienced scientist in using the UTB framework [24], to apply the data to the UTB framework constructs. The final conceptual framework was reviewed and approved by the Syilx peer researchers.

## Results

### Sample

A total 16 youth who identified as Syilx First Nation youth participated in this study. Youth ranged in age from 11 to 26, with the average age being 15.8. Most (62.5%) (10/16) of the sample was male. All participants were born in Canada. With the exception of one youth who had recently graduated high-school, the participants were all currently attending school, which ranged from grade 6 through to University. In relation to their social context, the majority of the sample lived in British Columbia and resided with 4 to 5 other individuals (68.75%;11/16 and 62.5%; 10/16 respectively). Most (81.25%) (13/16) of them had older peers. In relation to their tobacco-product use and exposure, many (56.25%) (9/16) youth had tried smoking and vaping, and most of them had friends who smoked (68.75%) (11/16) and vaped (87.5%) (14/16). Five (31.25%) youth reported living with someone who vaped, 50% (8/16) of youth reported that someone had tried to sell them vape juice, and 62.5% (10/16) of youth reported living close to a vape retail outlet.

We also examined social media use among youth. Most of the youth used Instagram (87.5%; 14/16), Snapchat (87.5%;14/16), YouTube (100%; 16/16) and Tik Tok (81.25%; 13/16) sometimes or all the time. Facebook (50%; 8/16) and Messenger (56.25%; 9/16) followed in popularity. Many youth reported seeing e-cigarette advertisements sometimes or all the time on Instagram (56.25%; 9/16), Snapchat (43.75%; 7/16), YouTube (37.5%; 6/16) and Tik Tok (43.75%/16).

### Qualitative findings

The findings were grouped under three major categories, and include: context of vaping among Indigenous youth, UTB determinants to vaping decision-making, and suggestions for prevention messages. Each of these categories have embedded themes with exemplary quotes. Each major finding is associated with the number of participants endorsing that finding to indicate saturation. Given shared perceptions between both those who vaped and those who did not vape, their data was pooled together. See Fig. 1 for a conceptual framework that presents how each of these findings relate to one another, and to the UTB framework.

### Context of vaping among indigenous youth

#### Disproportionate impact of the tobacco industry (*n* = 9)

First Nation youth agreed that vaping, although linked to tobacco, did not hold any spiritual value. They explained that vaping is too modern and does not have that historical and traditional connection in the same way as tobacco. Conversely, youth described e-cigarettes as another product that further emphasizes how Indigenous youth are disproportionately impacted by the tobacco industry. They explained that First Nation youth, especially those on reserves, have limited access to vaping both physically and financially. They cautioned that this lends to the purchase of non-reputable and cheaper products, ultimately lending to the use of more harmful vaping devices by First Nation youth.

*Not to be stereotypical. But I mean, like most indigenous youth, their family is poor. So I mean, like, they don't exactly have the money to buy the vape themselves. So they'll have to buy some off brand, that's cheaper (Sharing Circle).*

#### Vaping as colonialism (*n* = 12)

Youth also described vaping as being directly opposed to traditional ceremony, and as something that is in conflict with their cultural values. They explained how important it is to feel connected to their community, family, and culture, and the value of role-modeling within their band and community. Connection and role-modeling came up frequently during the interviews and the sharing circle, which they described as very important to their Indigenous culture. According to youth, vaping conflicted with these values. For example, several youth described the importance of being a role model in the community, and they perceived vaping as something that would detract from that.

*Community, I’m thinking…of Indigenous communities so, making sure that people are sticking to like their ways and values… (Interview, 1012).*

*[I want to be] like this good role model so that they’re like, “Oh I want to be like her she doesn’t vape, she participates in culture, and she does her best to connect with her community.” That’s the kind of person I want to be. I don’t want to be that kind of person where it’s like, “Oh she vapes, oh she might get into drinking.” (Interview 1003)*

### UTB determinants to vaping decision-making

Themes related to UTB determinants to vaping decision-making among Indigenous youth are detailed in Tables 2 and 3. Table 2 provides a list of individual determinants that influence intentions to vape and determinants that translate intentions to vape to decision to vape. Table 3 provides a list of individual determinants that influence intentions to not vape and determinants that translate intentions to vape to decision to vape.

**Table 2:** UTB determinants to vaping

Theme (UTB construct)	Exemplary Quotes	Saturation (***n*** = 16)
***Individual determinants that influence intentions to vape***		
Vaping helps you cope (behavioral belief)	It all came to this point where its like “Wow, this is really cool” and “I see this as something to get my mind off stuff.” (Interview, 1011) I think they choose to vape to calm down maybe and to relieve stress and anxiety because of things going on in the home, bad things. (Interview, 1002)	14
Vaping makes you cool (behavioral belief)	It could also be like “oh their doing it, and their cool” so its like uh, peer pressure kind of thing. And they feel pressured like “oh if I don’t do this, then I’m not cool.” And that’s something I’ve noticed a lot through out high school is like the cool factor (Interview, 1003)	15
Network approval through vaping (normative belief)	I don’t think anyone really does [approve]. No one like advocates for it or anything…its not like their like, “Yeah, do vape!”. They’re kind of like “Yeah, it’s kinda bad for me, but I do it.” (Interview, 1001) I would say my friends approve of it because I also know that they do that [vape]. (Interview, 1012)	13
***Social panic (split-second decision making)***	I think with most people it’s just social panic, like, kind of freeze in the moment….you think you’ll be prepared for it when somebody offers you [a vape], but then it really makes you freeze. And you don’t really…think about it to an extent….you just [vape] in the moment. (Sharing Circle)	**10**
***Determinants that facilitate the translation of intentions to vape to decision to vape***		
Lack of school policy enforcement (environment)	It’s just not enforced….Its just like, like a vague threat that doesn’t really actually [happen]. It’s like “This could happen to you” but it doesn’t. (Interview, 1001)	12
Access to vaping (environment)	[It’s easy to vape] if my friends have it and their like, “Hey I got this off of somebody” or something like that. (Interview, 1008) I think more accessible cause there’s not like a smell really and yea it’s easier to hide. So I feel like it’s the go to substance abuse. (Interview, 1012)	5

**Table 3:** UTB determinants to not vaping

Theme (UTB Construct)	Exemplary Quotes	Saturation (***n*** = 16)
***Individual determinants that influence intentions to not vape***		
Vaping is bad for your lungs (behavioral belief)	Not many my peers attending gym class anymore because they become short breath. Because they kept using their devices- coughing, some of them getting sick, because they took too much nicotine. (Sharing Circle)	14
Vaping is like “digging yourself into a hole” (behavioral belief)	I was just getting more and more addicted to it and I was becoming sad, gaining depression. And I had to dig myself out of that hole with friends and family and having conversations about how I wasn’t feeling okay. I had to go outside of my comfort zone and talk about how I was weak when it came to addiction. (Sharing Circle)	10
Family/community connectedness (normative beliefs)	It brings me hope and it makes me happy because participating in culture and connecting with my community means a lot to me. And I feel like I learned more of not only the Nation I’m from, but also more about who I am as a person. Cause like I feel like culture plays a huge role in finding out who you are as a person and what you can do not only to connect with your community, but also other communities within your Nation. That’s how I think of it. (Interview, 1003)	13
Family values and beliefs (normative beliefs)	When someone offers me something like that, I tend to think about what my parents have told me…I tend to think about what my mother tells me and what my dad tells me of what happens with that kind of stuff. (Interview, 1011)	5
Engaging in culture (self-efficacy)	Another thing I’d pick up is my runners for the unity run and like going hiking. I’d rather go hiking around, running, dancing, and I’d be like, “No, I’m good, I’m an athlete, and I’ve got to keep my health in mind.” Because I’d rather participate in my culture and my community. (Interview, 1003)	12
Self-reflection (self-efficacy)	What I did to stop was [by] questioning myself and saying, “Why am I doing this? What am I gaining? Am I gaining anything?” “No”, was the answer. I was getting nothing. (Sharing Circle)	11
Avoidance/resistance strategies (self-efficacy)	I always try to get my friends to maybe draw, just talk, or find games that we can do together… because I mean, it was really a red flag whenever they asked to go outside, because I mean, that’s where they mostly did [the vaping.] (Sharing Circle)	10
***Determinants that facilitate the translation of intentions to not vape to decision to not vape***		
Access to supportive adults/band (environment)	And I had, I had to find a way out [of vaping] and my way out was by [having conversations] with people I can trust. That was my way to stop vaping before. (Sharing Circle)	12
School policies (environment)	I think a school is supposed to be like a good role model. I guess not [a place] to do bad things. (Interview, 1008) [School policies] encourage many people not to bring any vapes, and encourage people to do more learning than vaping. Schools shouldn’t be the place where you have habits like that. (Interview, 1002)	5

### Suggestions for prevention messages

#### Need to resonate with why indigenous youth vape (*n* = 12)

Youth agreed that current prevention strategies were not resonating with youth. They said that prevention efforts are not acknowledging what is driving the behavior in the first place. One youth mentioned that many youth are vaping to help them cope. In this case, confiscating vapes is not helping them:

All I can say is that the methods that we have now for prevention of youth using vapes, drugs, this and that are just not really working. If I'm gonna be honest, at least in my general area, because I see people do it more and more and more because they get more and more annoyed at the fact that their things are getting taken, and they get more stressed, and have anxiety, and then they go back to it, and then they get into that loop of, “I need something to help me with this problem”…that's their only way of coping. (Sharing Circle)

The youth emphasized the need for prevention efforts that resonate with youth, not only such that they address the underlying factors that lend to vaping uptake, but also leverage the strengths and resiliency of Indigenous youth to affirm intentions to stay away from vaping.

#### Need to be where youth are at (*n* = 12)

Youth explained that message content and delivery channels need to meet youth where they are at. They said that prevention needs to be a focus within their community, as well as on social media channels that they frequently use (e.g., Instagram). Messages that would resonate with them should include a focus on helping youth self-reflect (e.g., asking yourself “why?”, “what’s the point?”, “what do you gain”, “how does this impact your culture?”, “how does this impact your role modelling?”), describing things that they could buy instead of vaping, helping them build skills to manage situations of social panic, have peers share their personal experiences with vaping, and have endorsement by celebrities/influencers.

## Discussion

This analysis aimed to identify factors that influence the decision to vape among Indigenous youth. While Indigenous scholars have called for Indigenous-specific evidence around youth tobacco use behaviors given their unique history and experiences [3, 4, 6](Seo & Chang 2021; Waa et al., 2019; Waa et al.., 2020; Thompson et al., 2020; Thompson & Thompson, 2020)), we have yet to understand the interplay between Indigenous youth contexts and salient behavioral constructs surrounding the decision to vape. We explored this by grounding a novel behavior change theory, the UTB, in a culturally responsive approach, and using Indigenous-led research methods.

The UTB surfaced as a flexible model that can be integrated with frameworks and contexts that are true to the communities in which they are situated. In this study, we found that a history of colonialism and being disproportionately impacted by the tobacco industry played a key role in what behavior change constructs surfaced as important factors in driving the decision to vape among Indigenous youth, and how Indigenous youth need to be supported. For example, we found that the behavioral belief that vaping helped youth cope with stressors, such as a home environment that entailed violence or substance use, as a result of colonialism, was a key driver in uptake among Indigenous youth. Conversely, connectedness with their community and their culture (the opposite of colonialism) (normative beliefs), was key to prevention of vaping uptake, and further supported other protective constructs (e.g., self-efficacy) in relation to vaping. Being able to ground the UTB constructs within the participant contexts is a major strength of this model and demonstrates its potential for use in other community contexts and with other demographics, enhancing the ability to develop and tailor behavior change interventions that are theoretical and evidence-based.

One of the key findings of this study is the link between vaping and the effects of colonialism. That youth were turning towards vaping as a result of intergenerational trauma and negative impacts of colonialism points towards an important history that underpins health risk behaviors among Indigenous youth, like vaping. While coping, curiosity, and peer influences played a role in their uptake, similar to their non-Indigenous peers [1], the findings reinforce the need to pay attention to another layer, a history that underpins these determinants of vaping. These findings build on previous research that describes how the erosion of social structures and intergenerational connectedness as a result of colonialism have directly and indirectly impacted the use of commercial tobacco products [7, 32–34]. The tobacco industry has capitalized on this erosion to promote their products, and are now targeting Indigenous people with alternative nicotine delivery systems (including e-cigarettes) as a way to make amends for past harms caused by cigarette marketing, essentially as a form of reconciliation [4, 6, 7]. Because Indigenous youth are more likely to be exposed to risk factors associated with substance use [35], which puts them at higher risk for using e-cigarettes [7], allowing marketing of these products to Indigenous people opposes truth and reconciliation efforts. Even more so, by not investing resources and engaging Indigenous people to specifically protect Indigenous youth may also be viewed as complacent colonization. Having a clear plan of investment, implementation, and evaluation of efforts to protect Indigenous youth from the harms of the tobacco industry is a necessary step towards truth and reconciliation [13].

Drawing on their cultural strengths (e.g., wisdom from elders, support from their community/band, engaging in cultural activities, viewing themselves as a role model in their community) were important to helping Indigenous youth think through their decision-making around vaping. Researchers have found that connection with their cultural values and traditions is an important way to combat the effects of colonialism, allowing Indigenous people to heal [16, 18, 19]. Our findings extend this research by revealing that culture, community connectedness, and traditional ceremony and values are important to Indigenous youth of today, and are essential to protecting them from engaging in health risk behaviors, and specifically vaping. In this regard, prevention messaging needs to reflect these values, which can only truly be done by continuing to engage Indigenous youth in the planning and development of prevention messages to ensure that they resonate with their contexts and value systems [18].

The findings provide some very tangible directions for developing prevention messaging that resonates with the contexts of Indigenous youth. A number of determinants point to what types of content should be included in these messages. For example, messages that leverage the cultural strengths of Indigenous youth (e.g., importance of being a role model), promote self-efficacy (e.g., suggest strategies and refusal skill building to deal with situations that might result in the split-second decision to vape), and reinforce their community supports (e.g., elders) hold significant promise in resonating with Indigenous youth. The unanimous agreement among the youth participants that social media would be the most desirable mode of delivery for these messages builds on previous research findings that this is the most effective way to reach today’s youth [36]. However, in light of the findings, a critical aspect in future development of prevention messages is the engagement of Indigenous youth throughout all aspects of research.

The findings also hold policy implications. In particular, the accessibility of vaping products among the participants was noteworthy. Not only did the majority of youth describe proximity to a vape store, in addition to access through peers or older individuals in their lives (e.g., older friends, parents, etc.), but they also described a lack of regulations (vaping indoors) and policy enforcement (school policies) as enabling easy access to vaping. What these findings indicate is that more attention needs to be paid to policy implementation at government, community, and school levels. On a government level, policy efforts are needed to more effectively reduce youth exposure to vape advertisements and retail. Policies around retail locations are also needed. For example, proximity to schools, recreation centers, and Indigenous communities should be considered. On a community level, regulations on the places where vaping is permitted need to be examined. It is important that a community enforces regulations that strengthen the self-determination and decision-making agency among Indigenous youth, such as through limiting or banning public indoor use. On a school level, the findings indicate a clear need to enforce vaping policies.

## Limitations and strengths

There are some limitations and strengths that must be noted in this study. First, the findings represent the voices of Syilx First Nation youth, and may not be transferrable to other Nations. It is also a strength of this study because the findings represent a united voice, and other Nations can compare how their values and beliefs might align. In addition, the findings represent the voices of urban Indigenous youth, and the perspectives and experiences of youth living on reserves may differ. Further, recruitment procedures may have resulted in voices being excluded. For example, by recruiting youth participating in an Indigenous summer camp program, the findings reflect the views of youth who are already engaging with their culture and see this as an important aspect of their identity. Finally, due to the nature of the sharing circle, there may have been alternative views not voiced in the presence of peers. However, this Indigenous method of data collection is also a major strength of the study because it is a method that is familiar to the youth, and is grounded in communication traditions that are important to their culture. Another major strength of this study is that it employed a culturally responsive approach, and a comprehensive behavior change theory to understand factors that influence vaping decision-making among Indigenous youth. This provides future researchers with a strong theoretically-informed evidence base from which to address the issue of vaping among Indigenous youth.

## Conclusions

The results of this study identify the most pertinent factors that underpin decision-making around vaping among Indigenous youth. Through the use of the UTB and a culturally-responsive approach, the findings provide a strong contextual and theoretical base for understanding these factors and providing a way forward. To effectively address vaping among Indigenous youth, continued engagement of Indigenous youth in planning, developing, implementing, and evaluating both prevention and policies efforts are a necessity.

## Data Availability

The datasets generated and analyzed during the current study are not publicly available due to personally identifiable data from the participants.
